# Effect of Cyclic Dynamic Compressive Loading on Chondrocytes and Adipose-Derived Stem Cells Co-Cultured in Highly Elastic Cryogel Scaffolds

**DOI:** 10.3390/ijms19020370

**Published:** 2018-01-26

**Authors:** Chih-Hao Chen, Chang-Yi Kuo, Jyh-Ping Chen

**Affiliations:** 1 Department of Chemical and Materials Engineering, Chang Gung University, Kwei-San, Taoyuan 33302, Taiwan; chchen5027@gmail.com (C.-H.C.); onesky1997@hotmail.com (C.-Y.K.); 2 Department of Plastic and Reconstructive Surgery and Craniofacial Research Center, Chang Gung Memorial Hospital, Linkou, Chang Gung University School of Medicine, Kwei-San, Taoyuan 33305, Taiwan; 3 Research Center for Chinese Herbal Medicine, Research Center for Food and Cosmetic Safety, College of Human Ecology, Chang Gung University of Science and Technology, Kwei-San, Taoyuan 33302, Taiwan; 4 Department of Materials Engineering, Ming Chi University of Technology, Tai-Shan, New Taipei City 24301, Taiwan

**Keywords:** cryogel, mechanical stimulation, dynamic culture, compressive loading, cartilage-tissue engineering, chondrocytes, adipose-derived stem cells

## Abstract

In this study, we first used gelatin/chondroitin-6-sulfate/hyaluronan/chitosan highly elastic cryogels, which showed total recovery from large strains during repeated compression cycles, as 3D scaffolds to study the effects of cyclic dynamic compressive loading on chondrocyte gene expression and extracellular matrix (ECM) production. Dynamic culture of porcine chondrocytes was studied at 1 Hz, 10% to 40% strain and 1 to 9 h/day stimulation duration, in a mechanical-driven multi-chamber bioreactor for 14 days. From the experimental results, we could identify the optimum dynamic culture condition (20% and 3 h/day) to enhance the chondrocytic phenotype of chondrocytes from the expression of marker (*Col I*, *Col II*, *Col X*, *TNF-α*, *TGF-β1* and *IGF-1*) genes by quantitative real-time polymerase chain reactions (qRT-PCR) and production of ECM (GAGs and Col II) by biochemical analysis and immunofluorescence staining. With up-regulated growth factor (*TGF-β1* and *IGF-1*) genes, co-culture of chondrocytes with porcine adipose-derived stem cells (ASCs) was employed to facilitate chondrogenic differentiation of ASCs during dynamic culture in cryogel scaffolds. By replacing half of the chondrocytes with ASCs during co-culture, we could obtain similar production of ECM (GAGs and Col II) and expression of *Col II*, but reduced expression of *Col I*, *Col X* and *TNF-α*. Subcutaneous implantation of cells/scaffold constructs in nude mice after mono-culture (chondrocytes or ASCs) or co-culture (chondrocytes + ASCs) and subject to static or dynamic culture condition in vitro for 14 days was tested for tissue-engineering applications. The constructs were retrieved 8 weeks post-implantation for histological analysis by Alcian blue, Safranin O and Col II immunohistochemical staining. The most abundant ectopic cartilage tissue was found for the chondrocytes and chondrocytes + ASCs groups using dynamic culture, which showed similar neo-cartilage formation capability with half of the chondrocytes replaced by ASCs for co-culture. This combined co-culture/dynamic culture strategy is expected to cut down the amount of donor chondrocytes needed for cartilage-tissue engineering.

## 1. Introduction

Cartilage is a unique connective tissue capable of enduring long-term tensile stress, compressive stress and heavy loads in the human body. The basic function of cartilage, particularly articular cartilage, is to lubricate the joints and absorb applied shock. However, the advance of age, disease and sports injuries can trigger metabolic disorders and structural damage in articular cartilage; in severe cases, these can even lead to the complete loss of the lubricating and shock-absorbing functions. Cartilage is comprised of chondrocytes that constitute approximately 10% (*w*/*v*) of the cartilage tissue. Because chondrocytes are only present in small amounts, cartilage is devoid of blood vessels and nerves, and the migration of chondrocytes to damaged areas for tissue repair is very difficult. In other words, once the cartilage is damaged, it has extremely limited self-repair and regeneration capabilities [1]. This poses clinical problems that urgently require a proper solution. The clinical practice for damaged articular cartilage repair includes microfracture, autologous chondrocyte implantation and mosaicplasty [2], which are autografting techniques that use cartilage from other parts of the body to repair damaged areas. However, these practices cause additional damage; and the repair is also subject to numerous restrictions, such as the required mechanical strength, and the amount, shape and size of the available cartilage tissue for replacement. To counter such shortcomings, tissue-engineering technique may offer a promising approach for further development. Cartilage tissue engineering involves the use of a 3D porous biomaterial scaffold with sufficient space and mechanical strength for chondrocytes to grow and secrete extracellular matrix (ECM), eventually forming a functional and solid tissue to repair or rebuild cartilage defects [3,4]. However, cartilage tissue engineering faces tremendous challenges, such as the difficulty cultivating large amounts of functional chondrocytes in vitro and insufficient secretion of ECM. Nevertheless, the literature suggests that physical stimulation in a 3D environment can promote cell proliferation and ECM secretion of chondrocytes [5]. Thus, the use of dynamic culture in a bioreactor with scaffolds that mimic the nature of an ECM microenvironment of chondrocytes for cartilage tissue engineering development is a topic worthy of exploration.

A number of cell and tissue types within the body demonstrate sensitivity to mechanical forces by regulating their gene expressions [6,7]. The specific changes of mRNA levels are mostly dependent on cell type and mechanical stimulus. Articular chondrocytes offer an example of such force-sensitivity, as tissue from which these cells originate from, articular cartilage, largely functions as a mechanical tissue and serves to absorb and evenly distribute compressive loads between long bones. Indeed, articular cartilage routinely experiences stress amplitudes of 10–20 MPa and compression up to 45% [8]. For articular cartilage, compression amplitudes have been reported to be higher than 13% for daily movements, while loadings were from 0.5–7.7 MPa for cartilage in the weight-bearing joints of the hip and knee [9]. Nonetheless, chondrocytes appear to be very selective toward mechanical stimuli, as in vitro dynamic loading was found to increase proteoglycan and protein synthesis by chondrocytes, but static loading to decrease their production [10,11]. Therefore, it has been generally recognized that dynamic loading within appropriate ranges of frequency, load and strain is more beneficial to cartilage than sustained static loading, which may be a useful tool for tissue engineering of articular cartilage. In addition, the mechanical loading experiments have been mostly carried out in bioreactors using uniaxial compression. The potential benefits of applying dynamic compression on ECM synthesis rates by chondrocytes have been demonstrated in studies using chondrocytes seeded on a range of scaffolds, including agarose hydrogels [12], self-assembling peptide hydrogels [13] and macroporous polylactic acid/polyglycolic acid (PLA/PGA) scaffolds [14].

We have previously reported the preparation of an elastic macroporous gelatin/chondoitin-6-sulfate/hyaluronan/chitosan cryogel scaffold with high porosity, large pore size, and unique mechanical properties (high elasticity and total recovery from large strains) for cartilage tissue engineering [15]. Other than showing comparable biomechanical properties to those of cartilage, the cryogel scaffold also showed superior recovery property from large strains (30%) and the same hysteresis curves during successive loading/unloading cycles (3200 cycles). These unique features, i.e., recovery from large strains and absorbing impacts without permanent damage, make cryogel a most suitable candidate as a 3D scaffold for dynamic culture in a bioreactor subject to constant loading/unloading cycles.

On the other hand, several in vitro cell-culture reports have shown that cartilage tissue or isolated chondrocytes influence other chondrocytes or mesenchymal stem cells (MSCs) via soluble paracrine factors [16,17]. The co-culture of primary chondrocytes with in vitro-expanded de-differentiated chondrocytes reverses the profile of de-differentiation [17]. Furthermore, the mechanism to direct in vivo ectopic chondrogenesis of MSCs was reported to be due to paracrine signaling from chondrogenic factors produced by chondrocytes [18]. The co-culture system that could be used to study the differentiation mechanism of stem cells is determined by a tissue-specific niche. Therefore, we postulated that incorporation of chondrocytes into a cyclic compression bioreactor may improve the functional maturation of adipose-derived stem cells (ASCs) toward tissue-engineered cartilage.

In the present study, we first prepared a highly elastic cryogel scaffold synthesized from gelatin, chondroitin-6-sulfate, hyaluronan and chitosan as a 3D scaffold to provide chondrocytes a stable and ideal physiological environment during dynamic culture. Gene expression, cell proliferation and ECM production were studied in the scaffold by culturing porcine chondrocytes in the cryogel scaffold subject to different cyclic dynamic compression loading conditions. The best dynamic culture condition to enhance chondrocyte phenotype was chosen for co-culture of porcine chondrocytes and ASCs in the scaffolds. Subcutaneous implantation of cells/scaffold constructs were used to test the ectopic neo-cartilage tissue-formation capabilities of the mono-culture and co-culture groups after static and dynamic culture in vitro.

## 2. Results

### 2.1. Effects of Cyclic Compressive Loading on Gene Expression of Chondrocytes in Cryogel Scaffolds

**Figure 1:**
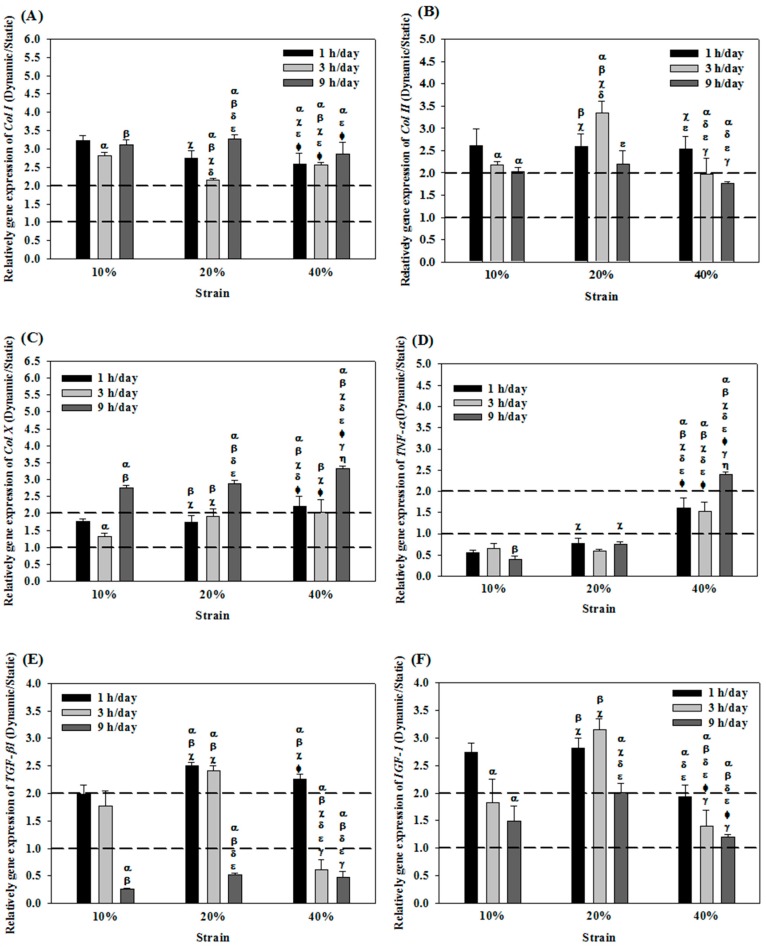
The relative gene expression when chondrocytes were cultured in cryogel scaffolds under different cyclic compressive loading conditions. The expression of *Col I* (**A**); *Col II* (**B**); *Col X* (**C**); *TNF-α* (**D**); *TGF-β1* (**E**) and *IGF-1* (**F**) for cells in dynamic culture at 1 Hz was normalized to those in static culture at day 14. The groups under dynamic culture with 10% (40%) strain and 1 h/day (3 h/day) duration are termed D11 (D43) etc. α *p* < 0.05 with D11; β *p* < 0.05 compared with D13; χ *p* < 0.05 compared with D19; δ *p* < 0.05 compared with D21; ε *p* < 0.05 compared with D23; Φ *p* < 0.05 compared with D29; γ *p* < 0.05 compared with D41; η *p* < 0.05 compared with D43. *Col II*: Type II collagen; *Col I*: Type I collagen; *Col X*: Type X collagen; *TNF-α*: Tumor necrosis factor-α; *IGF-1*: Insulin growth factor-1; *TGF-β1*: Transforming growth factor-β1.

When chondrocytes in cryogel scaffolds were subjected to cyclic compressive loading during dynamic culture, the expression of *Col I* was more than twice as high as that in static culture (Figure 1A). At 20% strain and 3 h/day duration (D23), *Col I* exhibited the lowest expression, which was 2.1-fold compared with static culture. The expression of *Col II* after mechanical stimulation was also up-regulated compared with static culture but with distictive feature from *Col I*. The D23 group showed the highest value, which was 3 times that in static culture increase. The D43 and D49 groups showed the lowest values, which were less than 2 times as high when compared with that from static culture (Figure 1B). Notably, the strain used had no influence on *Col I* and *Col II* expressions when the duration was fixed at 1 h/day. The expressions of *Col X* was consistently higher when subject to dynamic culture with 9 h/day generated higher values than 1 or 3 h/day and the highest up-regulation of *Col X* (3.3 fold) was shown at 40% strain (D49) (Figure 1C). For *TNF-α*, a reverse trend was observed as the gene expression subject to cyclic compressive loading was generally lower than those produced through static culture, except when strain = 40% (Figure 1D). These results implied that we could adopt properly tuned cyclic compressive loading during dynamic culture to control the expression of the cartilage-specific maker gene *Col II* and the negative regulator genes *Col I*, *Col X* and *TNF-α*. It is suggested that the optimal dynamic culture condition could be 20% strain and 3 h/day stimulation duration (D23). Further analysis of the positive regulator genes *TGF-β1* and *IGF-1* also endorsed the choice of D23, as both growth factor-related genes showed the highest expression levels at this setting (Figure 1E,F). Overall, analysis of gene-expression levels successfully identified a setting that could provide the best option for the dynamic culture of chondrocytes in the cryogel scaffold when subject to cyclic compresson loading in the bioreactor.

### 2.2. Effects of Cyclic Compressive Loading on DNA, Glycoaminoglycans (GAGs) and Type II Collagen (Col II) Contents of Chondrocytes in Cryogel Scaffolds

**Figure 2:**
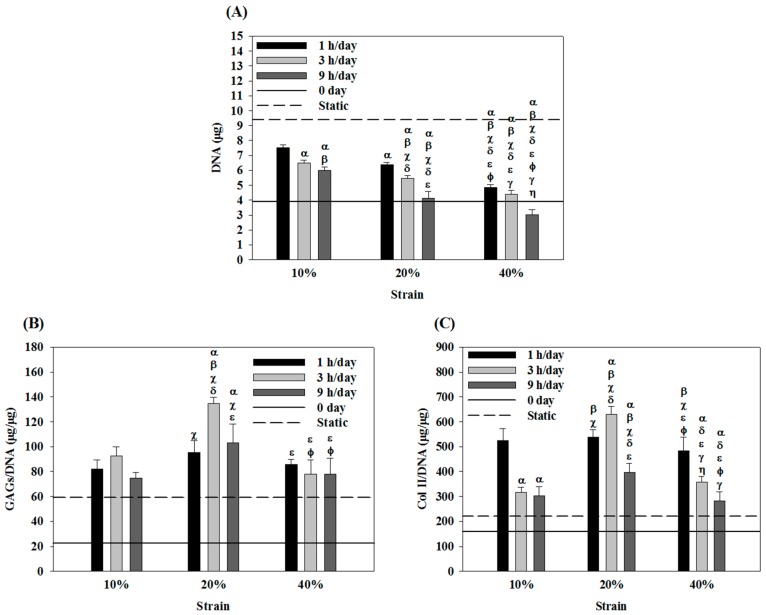
The proliferation and extracellular matrix (GAGs and Col II) production when chondrocytes were cultured in cryogel scaffolds under different cyclic compressive loading conditions. The DNA (**A**), GAGs/DNA (**B**) and Col II/DNA (**C**) contents of cells dynamically cultured for 14 days at 1 Hz were shown and compared with day 0 (solid lines) and static culture (dotted lines) for 14 days. The groups under dynamic culture with 10% (40%) strain and 1 h/day (3 h/day) duration are termed D11 (D43) etc. α *p* < 0.05 with D11; β *p* < 0.05 compared with D13; χ *p* < 0.05 compared with D19; δ *p* < 0.05 compared with D21; ε *p* < 0.05 compared with D23; Φ *p* < 0.05 compared with D29; γ *p* < 0.05 compared with D41; η *p* < 0.05 compared with D43. GAGs: Glycoaminoglycans; Col II: Type II collagen.

Under mechanical stimulation, the growth of chondrocytes in the cryogel scaffold was found to be slower than that in static culture (Figure 2A). Notably, an increase in strain or duration synergistically decreased chondrocyte cell number at the end of the culture period. At the highest strain (40%) and duration (9 h/day), the number of chondrocytes at day 14 was even less than the starting cell number (0 day), suggesting that cyclic compressive loading either with higher strain or prolonged duration may be detrimental for the proliferation of chondrocytes in the cryogel scaffold. Therefore, the lowest strain (10%) and the shortest duration time (1 h/day) may be suggested for dynamic culture considering the proliferation of chondrocytes.

Considering GAGs production, the 20% strain groups consistently produced higher normalized GAGs value (GAGs/DNA) (Figure 2B). Moreover, at 10% or 20% strain, the highest GAGs/DNA value occurred when duration = 3 h/day. In contrast, the GAGs/DNA value was independent of duration at 40% strain. Taken together, the best production of GAGs by chondrocytes in the cryogel scaffold under cyclic dynamic loading could be suggested at 20% strain and 3 h/day duration (D23), which is the same setting determined from previous gene-expression analysis. At this condition, the GAGs production was 2.3 times that in static culture.

For Col II production on a per cell basis, the Col II/DNA value was not affected by the strain when duration = 1 h/day (Figure 2C). Nonetheless, further increases to 3 or 9 h/day resulted in strain-dependent Col II production, with the intermediate strain (20%) giving the highest value of Col II/DNA. Therefore, as with GAGs production, the D23 group could be deemed as the best setting for considering Col II production, where the Col II/DNA value was 2.9 times that in static culture.

**Figure 3:**
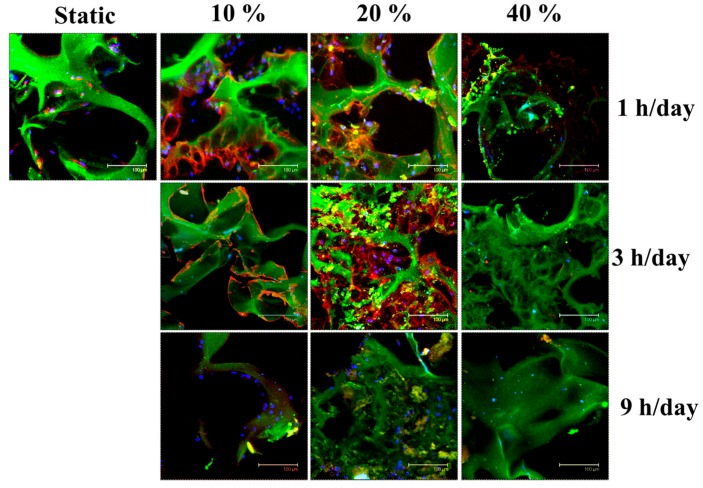
Immunofluorescence staining of chondrocytes in the cryogel scaffolds in static or dynamic culture (frequency = 1 Hz) with different cyclic compressive-loading conditions. Blue: cell nucleus (DAPI); red: Col II (Cy 3); green: gelatin (FITC); scale bar = 100 µm. DAPI: 4′,6-diamidino-2-phenylindole; Cy 3: Cyanine 3; FITC: Fluorescein isothiocyanate.

Further confirmation of the effects of mechanical stimulation on Col II production could be visualized from the immunofluorescence staining images of the cells/cryogel constructs (Figure 3). The difference in Col II signal density (in red) was consistent with the values reported quantitatively in Figure 2C, with the strongest Col II fluorescence signal shown at 20% strain and 3 h/day duration time. Overall, using cyclic compressive loading to stimulate chondrocytes mechanically in cryogel scaffolds during dynamic culture in the bioreactor could substantially improve the maintenance of the chondrocyte phenotype compared to static culture, considering both gene expression and ECM production and the best settings chosen for compressive dynamic loading should be the intermediate values of both strains (i.e., 20%) and duration (i.e., 3 h/day).

### 2.3. Effects of Cyclic Compressive Loading and Co-Culture on Gene Expression of Cells in Cryogel Scaffolds

**Figure 4:**
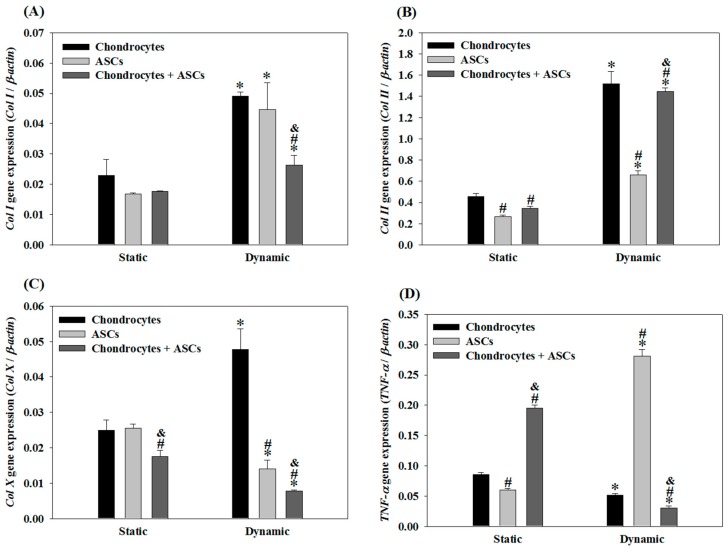
The gene expression of *Col I* (**A**); *Col II* (**B**); *Col X* (**C**) and *TNF-α* (**D**) when chondrocytes, adipose-derived stem cells (ASCs) or chondrocytes + ASCs were cultured in cryogel scaffolds for 14 days under static or cyclic compressive loading (frequency = 1 Hz, strain = 20% and duration = 3 h/day) conditions. Cell number: 10^5^ chondrocytes/scaffold for the chondrocytes group; 10^5^ ASCs/scaffold for the ASCs group; 5 × 10^4^ chondrocytes/scaffold and 5 × 10^4^ ASCs/scaffold for the chondrocytes + ASCs group. * *p* < 0.05 compared with the static group; # *p* < 0.05 compared with the chondrocytes group; & *p* < 0.05 compared with the ASCs group.

Using the optimal settings for the dynamic culture of chondrocytes, the gene-expression results indicated that *Col I* expression in dynamic culture was higher than that in static culture regardless of co-culture (chondrocytes + ASCs) or mono-culture (chondrocytes or ASCs) (Figure 4A). Nonetheless, the co-culture group exhibited significantly lower *Col I* expression than that in each mono-culture group when subject to compressive dynamic loading, which was ~65% that of the chondrocytes’ group. For *Col II*, the trend observed is contrary to *Col I*, as the gene expressions were significantly up-regulated by dynamic culture (Figure 4B).

Specifically, although *Col II* expression of the co-culture group was only 76% of the chondrocytes group under static culture, the expression level of *Col II* was substantially improved when subject to cyclic compressive loading. The *Col II* expression of the ASCs and the co-culture group was 43% and 95% that of the chondrocytes group, respectively. Most importantly, the chondrocytes + ASCs co-culture group did not show a significant difference from the chondrocytes group in *Col II* expression, albeit substituting half of the chondrocytes with ASCs. This observation suggested that cyclic compressive loading, which influences the gene expression and ECM production of chondrocytes (Figure 1 and Figure 2), may induce the differentiation of ASCs toward the chondrogenic lineage, as revealed from up-regulation of the chondrogenic marker gene *Col II* when co-cultured with chondrocytes. On the other hand, dynamic culture and co-culture also appeared to synergistically suppress the expression of *Col X* and *TNF-α*. Specifically, co-culture could reduce *Col X* expression to 16% (55%) that of chondrocytes (ASCs) and reduced *TNF-α* expression to 58% (11%) that of chondrocytes (ASCs) in dynamic culture (Figure 4C,D). The *Col X* and *TNF-α* expression levels of the co-culture group also reduced to 44% and 15% when compared with the values in static culture.

### 2.4. Effects of Cyclic Compressive Loading and Co-Culture on DNA, GAGs and Col II Contents of Cells in Cryogel Scaffolds

**Figure 5:**
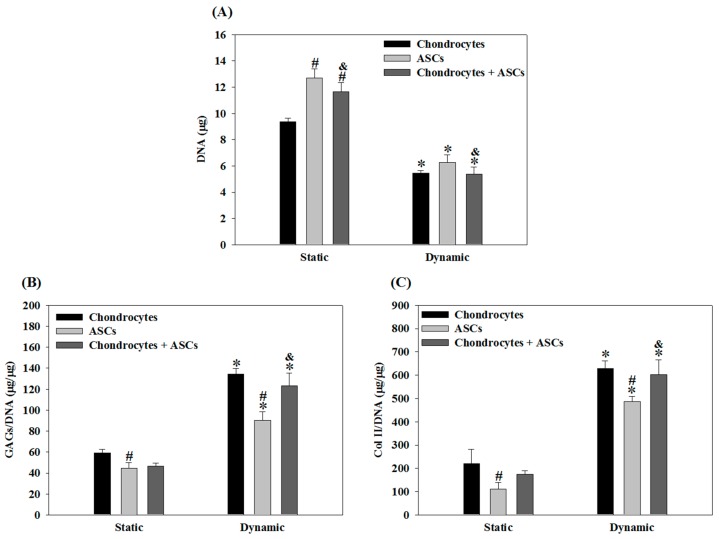
Proliferation and extracellular matrix (GAGs and Col II) production when chondrocytes, ASCs or chondrocytes + ASCs were cultured in cryogel scaffolds for 14 days under static or cyclic compressive loading (frequency = 1 Hz, strain = 20% and duration = 3 h/day) conditions. Cell number: 10^5^ chondrocytes/scaffold for the chondrocytes group; 10^5^ ASCs/scaffold for the ASCs group; 5 × 10^4^ chondrocytes/scaffold and 5 × 10^4^ ASCs/scaffold for the chondrocytes + ASCs group. (**A**) DNA; (**B**) GAGs/DNA; (**C**) Col II/DNA. * *p* < 0.05 compared with static; # *p* < 0.05 compared chondrocytes; & *p* < 0.05 compared with ASCs.

From DNA content, cell proliferation in all groups receiving cyclic compression was significantly reduced compared with static culture irrespective of mono-culture or co-culture. Specifically, cell number in dynamic culture was ~50% that in static culture for all groups (Figure 5A). Nonetheless, as with *Col II* expression, the dynamic culture and co-culture appeared to synergistically enhance GAGs production (Figure 5B). The GAGs/DNA content in the co-culture group under static culture and dynamic culture was 79% and 92% of chondrocytes, respectively. Also, no significant difference could be found for chondrocytes and chondrocytes + ASCs groups only when dynamic culture was used. Similarly, Col II production after compressive loading was higher than in static culture irrespective of culture conditions (i.e., mono-culture or co-culture) (Figure 5C). The Col II/DNA value in chondrocytes + ASCs group was 79% that in the chondrocytes’ group in static culture. This value increased to 96% in dynamic culture, with a significant difference from the ASCs group but without a significant difference from the chondrocytes’ group.

**Figure 6:**
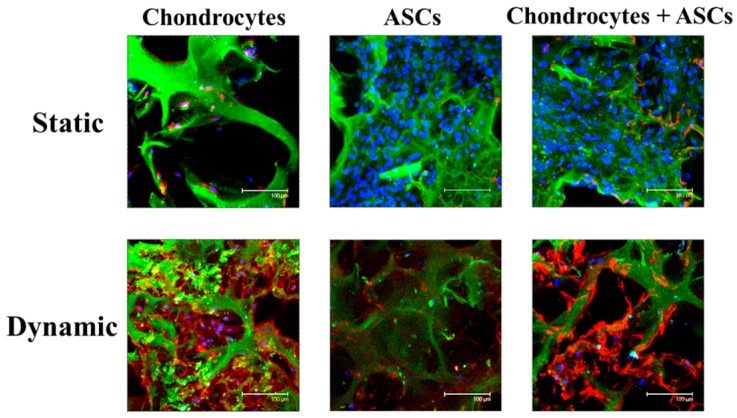
Immunofluorescence staining of cells/scaffold constructs when chondrocytes, ASCs or chondrocytes + ASCs were cultured in cryogel scaffolds for 14 days under static or cyclic compressive loading (frequency = 1 Hz, strain = 20% and duration = 3 h/day) conditions. Cell number: 10^5^ chondrocytes/scaffold for the chondrocytes group; 10^5^ ASCs/scaffold for the ASCs group; 5 × 10^4^ chondrocytes/scaffold and 5 × 10^4^ ASCs/scaffold for the chondrocytes + ASCs group. Blue: cell nucleus (DAPI); red: Col II (Cy 3); green: gelatin (FITC); Scale bar = 100 µm.

Immunofluorescence staining of Col II suggested that, as in the case of Col II/DNA, the production of Col II in the co-culture group was similar to the chondrocytes’ group under mechanical stimulation (Figure 6). As Col II is the most important ECM component of chondrocytes, substituting half of the chondrocytes with ASCs in conjunction with mechanical stimulation offers a viable strategy to reduce the number of chondrocytes needed to generate functional cartilage tissue.

### 2.5. In Vivo Subcutaneous Implantation of Cells/Scaffold Implantation

**Figure 7:**
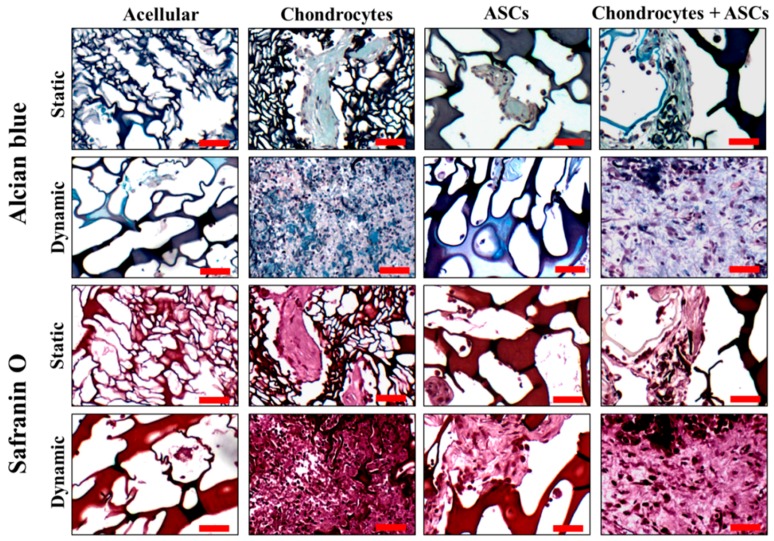
Alcian blue and Safranin O staining of implants eight weeks after subcutaneous implantation in nude mice. The cells/scaffold constructs were cultured in cryogel scaffolds for 14 days under static or cyclic compressive loading (frequency = 1 Hz, strain = 20% and duration = 3 h/day) conditions before implantation. Scale bar = 50 µm.

To reveal the ectopic cartilage formation potential of mono-cultured and co-cultured cells after in vitro culture in cryogel scaffolds under static or dynamic conditions, the cells/scaffold constructs were implanted subcutaneously in nude mice. The cell distribution and ECM synthesis were evaluated by Alcian blue and Safranin O staining of the retrieved samples. Eight weeks after implantation, a significant increase of GAGs components was evident from both Alcian blue and Safranin O staining compared with the acellular group (Figure 7). The staining intensity within the construct illustrated GAGs production in the chondrocytes + ASCs group was different from the ASCs groups but similar to the chondrocytes group under both static and dynamic culture conditions. Furthermore, the GAGs production of the construct cultured under dynamic condition was much higher than the same construct cultured statically prior to implantation. These trends are similar to the trends observed from the in vitro cell culture (Figure 5B), indicating the in vitro cultured constructs maintain the GAGs secretion capability after in vivo implantation.

**Figure 8:**
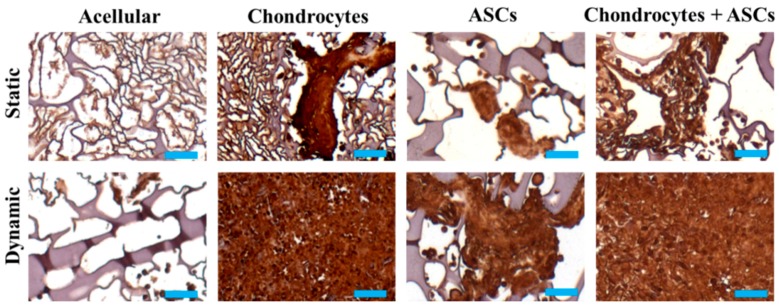
Immunohistochemical staining of Col II of implants eight weeks after subcutaneous implantation in nude mice. The cells/scaffold constructs were cultured in cryogel scaffolds for 14 days under static or cyclic compressive loading (frequency = 1 Hz, strain = 20% and duration = 3 h/day) conditions before implantation. Scale bar = 50 µm.

Immunohistochemical staining of Col II suggested that, similar to the trend observed for Col II/DNA in Figure 5C, enhanced Col II production after mechanical stimulation by cyclic compressive loading for cell-seeded groups (Figure 8). The staining intensity for the chondrocytes + ASCs group was similar to the chondrocytes group as for GAGs but much higher than the ASCs group, suggesting comparable ectopic neo-cartilage tissue formation capability of the constructs for the co-culture group using half of the chondrocyte cell number.

## 3. Discussion

As the mechanical environment regulates the development and maintenance of native cartilage tissue, mechanical stimulation during chondrocyte culture, which applies forces to the tissue constructs analogous to those experienced in vivo, is a popular method to enhance the synthesis of cartilaginous ECM [19]. The 2D mechanical stimulation of chondrocytes was first shown to result in rapid intracellular activation of signaling cascades related to membrane hyperpolarization and ion channels’ regulation pathways, which led to the secretion of cytokines and growth factor [20]. To date, many studies have shown the positive effect of dynamic compressive loading on metabolism and ECM synthesis by chondrocytes, ultimately affecting the functional properties of the developed tissue [21,22]. Thus, the use of bioreactors for chondrocyte culture in 3D scaffolds through dynamic loading has become a critical topic in articular cartilage tissue engineering [23,24].

As the cellular response to mechanical signals is regulated by the properties of the scaffold, it is important to design 3D scaffolds that present the appropriate signals necessary to direct cell function, such as incorporation of specific cell-adhesion ligands. This is especially important for engineering tissues that require mechanical stimulation for proper development, such as cartilage. The arginine–glycine–aspartic (RGD) ligand in poly(ethylene glycol) (PEG) RGD hydrogel was shown to enhance cartilage-specific gene expression and ECM synthesis only when cultured under mechanical stimulation condition [25]. In a separate study, enhanced cell proliferation and proteoglycan synthesis were found when chondrocytes entrapped in agarose gels were subjected to dynamic compressive loading [26]. Thus, the specific cell adhesion ligand RGD can play an important role in mediating cellular response through integrin-mediated mechanotransductive processes in chondrocytes. The first requirement of specific cell adhesion ligands in the scaffold was met by gelatin in the cryogel as it contains abundant RGD sequences which are the cell attachment sites recognized by many integrins [27].

Additional consideration for the scaffolds used for dynamic compression loading will be the possibility to manipulate the intrinsic mechanical properties of the scaffolds to mimic those of the tissue intended to regenerate. This requirement was also met by choosing the gelatin/chondoitin-6-sulfate/hyaluronan/chitosan cryogel scaffold for cartilage tissue engineering as the scaffold was shown to have comparable intrinsic mechanical properties with those of cartilage [15]. Furthermore, the cryogel was found to have a superior recovery property from large strains, indicating the macroporous structure will be retained when subject to cyclic compression cycles [15]. This behavior could ensure the scaffold preserves the same architecture in transmitting consistent mechanotransductive force to chondrocytes in the cryogel scaffold during the compression cycles. A previous study in which scaffold architecture was found to determine chondrocyte response to externally applied dynamic compression also underlines the preferred choice of cryogel as the scaffold in our study [28]. To the best of our knowledge, this study represents the first to use cryogel as a 3D scaffold for the dynamic culture of cells in a bioreactor.

Collagen is the most abundant structural macromolecule in the ECM of cartilage, making up about 60% of its dry weight [29]. Col II represents 90–95% of the ECM collagen, with other types of collagen (types I, IV, V, VI, IX, and XI) representing a minor proportion. Specifically, the minor collagens help to form and stabilize the Col II fibril network. Nonetheless, Col I indicates fibrosis or de-differentiation of chondrocytes, whereas Col II represents the renaturation of chondrocytes and normal expression of their phenotype [30]. From Figure 1A,B, the expressions of *Col I* were independent of stimulation duration when subject to a high strain (40%), whereas those of *Col II* consistently declined with an increase in stimulation duration at both high (40%) and low (10%) strains. When subject to a moderate strain (20%) stimulation, no such trends could be observed for both *Col I* and *Col II*. This indicates that expressions of *Col I* and *Col II* are susceptible to the influence of both strain and stimulation duration. Some studies have noticed similar results, with some research even suggesting that the suppression of *Col I* could promote the expression of *Col II* [31]. Our study determined that when under the optimum dynamic loading condition (strain = 20%, duration = 3 h/day), a better maintenance of the chondrocytes’ phenotype could be achieved judging from the marked down-regulation of *Col I* and up-regulation of *Col II* simultaneously.

The *Col X* represents one of the mechanosensitive genes in cartilage [32]. Indeed, Col X is a marker for hypertrophic cartilage since its mRNA is up-regulated in hypertrophic chondrocytes [33]. Interestingly, *Col X* is also induced in articular chondrocytes during osteoarthritis [34]. As present experimental results suggest (Figure 1C), excessive stimulation duration (9 h/day) and strain (40%) could promote the expression of *Col X* and lead to hypertrophic chondrocytes. That dynamic culture of chondrocytes in cryogel scaffolds subject to cyclic compressive loading substantially up-regulated *Col I*, *Col II* and *Col X* compared with static culture corroborates previous findings [23]. Similar to *Col X*, *TNF-α* is an indicator of chondrocytes degeneration [35,36]. Apoptosis may be induced in both hypertrophic and non-hypertrophic chondrocytes by TNF-α [37]. In addition to stimulating cartilage degradation, TNF-α also inhibits synthesis of ECM by chondrocytes while cyclic tensile strain of low magnitude acts as an effective antagonist of TNF-α by augmenting the proteoglycan synthesis that is inhibited by TNF-α [38]. Figure 1D illustrates that excessive strain (40%) promotes the expression of *TNF-α*, whereas other strain settings suppress such expression to a value even less than that in static culture. Therefore, dynamic culture with low to intermediate strains (10% and 20%) may contribute to protecting chondrocytes from losing their phenotype.

As shown in Figure 1E,F, using cyclic dynamic compression at the optimized settings for the dynamic culture of chondrocytes in the cryogel scaffold (i.e., 20% strain and 3 h/day duration) also resulted in the highest expression levels of *TGF-β1* and *IGF-1*. IGF-1 is one of the important factors involved in the proliferation of chondrocytes while TGF-β is involved in chondrocyte differentiation [39]. TGF-β1 and IGF-1 are the main molecules considered to be anabolic for cartilage [40]. The synergistic actions of TGF-β and IGF-1 induced the expression of Col II and aggrecan genes in human articular chondrocytes [41]. Mechanical stress induced by centrifugal pressure could increase the expression of *IGF-I* mRNA in the early stage of chondrocyte culture [42]. IGF-I also stimulates ECM synthesis in articular chondrocytes [43]. Furthermore, IGF-I also maintain chondrocyte phenotype and prevent the cells from going into the hypertrophic stage [44].

The biochemical analysis shown in Figure 2 revealed that mechanical stimulation could suppress the growth of chondrocytes, which was consistent with previous findings [45,46]. However, the Col II/DNA and GAGs/DNA values were much higher under mechanical stimulation than static culture (Figure 2B,C). It can be postulated that suppressing the proliferation of chondrocytes can lead to different, and most likely higher, ECM production as reported for the static culture of chondrocytes [23]. Immunofluorescence staining of Col II (Figure 3), which could show the distribution and production of this protein by chondrocytes within the cryogel scaffold, was consistent with the quantitative determination of Col II (Figure 2C). Therefore, excessive strain (40%) and stimulation duration (9 h/day) can be inferred to be the cause of chondrocyte hypertrophic changes and degeneration; conversely, the optimal stimulation setting (strain = 20%, stimulation duration = 3 h/day) can effectively suppress the fibrosis of cartilage and promote cartilage-specific Col II production and matrix deposition in the ECM.

Similar to enhanced chondrogenic gene expression and GAGs synthesis in chondrocytes, many studies involving dynamic compression suggested a beneficial effect of mechanical loading for chondrogenesis of MSCs [47]. Chondrogenic differentiation of MSCs is induced by IGF-I but is enhanced when TGF-β1 and IGF-I and are used in combination [48,49]. Thus, the present study also tested the hypothesis that co-cultured chondrocytes + ASCs under mechanical stimulation could enable chondrocytes to secrete growth factors (i.e., TGF-β1 and IGF-1) that could induce the differentiation of ASCs toward the chondrogenic lineage. This approach will reduce both the amount of chondrocytes and the demand for additional growth factors in the cell-culture medium during in vitro cell culture [50]. As previous studies demonstrated that different mechanical stimulation settings during dynamic culture could control the excretion of growth factors that promote chondrogenesis by ASCs, the aim was to find an optimized mechanical stimulation condition that can control the gene expression of growth factors related to chondrocytes’ differentiation (*TGF-β1* & *IGF-1*) and promote the differentiation of ASCs into chondrocytes [30]. As results in Figure 1 suggested that 20% strain and 1 or 3 h/day duration markedly enhanced *TGF-β1* expression (Figure 1E) while *IGF-1* showed the highest expression at 20% strain and 3 h/day duration (Figure 1F), 20% strain and 3 h/day duration (D23) were also selected as the optimal setting for dynamic co-culture of chondrocytes + ASCs.

According to Figure 4B, the *Col II* expression of the chondrocytes + ASCs group during static culture was 76% that of the chondrocytes group but 1.3 times that of the ASCs group using the same cell number but substituting half of the chondrocytes with ASCs. This trend was consistent with previous studies and confirms co-culture strategy was beneficial for chondrogenic differentiation of ASCs [50]. For dynamic culture, the *Col II* expression of the co-culture group was 95% that of the chondrocytes’ group and 2.2 times that of the ASCs group, endorsing the up-regulation *TGF-β1* & *IGF-1* of chondrocytes under mechanical stimulation contributed to the enhanced differentiation of ASCs into chondrocytes.

It is also evident from Figure 4 that applying cyclic compressive loading to the chondrocytes + ASCs group could suppress the expression of *Col I*, *Col X* and *TNF-α,* which are negative genes related to chondrogenesis, rather than the chondrocytes’ or ASCs groups alone. Furthermore, only dynamic culture, but not static culture, could lead to significant down-regulation of those negative genes in the co-culture group from the chondrocytes’ or ASCs groups. Taken together, our results suggested that combining co-culture and cyclic compressive loading may prevent hypertrophy and apoptosis of chondrocytes and better maintain their phenotype for gene analysis.

Finally, biochemical analyses verified that mechanical stimulation could suppress cell proliferation regardless of co-culture or not but promote the production of Col II and GAGs (Figure 5). The secretion of Col II and GAGs of the co-culture group was over 90% and not significantly different from those of the chondrocytes group, albeit the same cell number was used but with only half chondrocytes. The immunofluorescence staining results in Figure 6 showed a remarkable increase of Col II production concomitant with the reduced cell number, confirming the trend observed in Figure 5 where Col II production enhancement and cell growth arrest were induced by the dynamic culture of chondrocytes + ASCs. Comparing dynamic and static culture of ASCs, our results in Figure 5 were also in line with previous findings that suggested a beneficial effect of dynamic loading for chondrogenesis of MSCs, as revealed from increases in Col II and GAGs production using a frequency of 1 Hz and 10% or 15% compression strain [51,52]. Overall, our study suggested that chondrocytes under dynamic compressive loading in the highly elastic cryogel scaffold can induce the chondrogenic differentiation of ASCs. Furthermore, incorporation of chondrocytes into a cyclic compression bioreactor system can improve the functional maturation of ASCs toward tissue-engineered cartilage.

To verify the strategy employed here, which combined dynamic culture and co-culture and is potentially feasible for cartilage-tissue engineering, a subcutaneous implantation model was used to assess the ectopic cartilage-formation capability of the cells/scaffold constructs cultured under the static and dynamic culture conditions in vitro. The morphology of the chondrocytes, the distribution of cells and ECM synthesis were studied by Alcian blue and Safranin O staining. As the implantation period reached eight weeks, a significant increase of the cell number was observed, and the distribution of cells appeared to be homogeneous (Figure 7). A distinguishable difference in the constructs’ cultures, statically or dynamically before implantation, was the cell density within the interstitial matrix, which was higher for the dynamic culture group. Although the static culture group had fewer cells than the dynamic culture group after a 7-day in vitro cell culture (Figure 5A), more cells in the constructs were found for the dynamic culture group 8 weeks post-implantation. We may ascribe this phenomena to the up-regulation of *IGF-1* during dynamic cell culture in vitro (Figure 1F) as IGF-1 is a key growth factor exerting its actions to stimulate the proliferation of chondrocytes [53]. The cells were also found to be more clustered around each other, which may allow more cell interaction to stimulate more production of ECM [54].

The function of GAGs within the ECM interstices is to provide swelling capability for the cartilage, which could endow the connective tissue with more compressive-loading resistance [55]. Histological staining by Safranin O and Alcian blue revealed GAGs production during the implantation period (Figure 7). The increase in staining intensity within the constructs illustrated increased GAGs production, demonstrating the filling of the cartilaginous tissue in the constructs. Overall, the distribution of GAGs was much higher both in the chondrocytes and chondrocytes + ASCs groups under mechanical stimulation than all other groups, indicating continued matrix deposition in vivo.

As one of the ECM components of cartilage, Col II plays an important role in chondrogenesis and characterizes the chondrogenic phenotype of regenerated cartilage indirectly [56,57]. Positive staining of this cartilage-specific marker was only evident from the cell-seeded scaffolds 8 weeks post-implantation. That the immunohistochemical (IHC) staining was found to be more widespread in the dynamic group suggests that mechanical factors did play a crucial role in the development of chondrogenesis before constructs implantation [9,58]. In addition, it was noted that the tendency of Col II secretion was more prominent in the chondrocytes and chondrocytes + ASCs groups (Figure 8). The similar Col II intensity from IHC staining of these two group also suggests that the ASCs had been successful induced by chondrocytes for chondrogenesis. However, the optimal ratio of ASCs and chondrocytes and the exact mechanisms mediating cell-to-cell interactions during co-culture require further investigations for future clinical application.

## 4. Materials and Methods

### 4.1. Materials

Gelatin (type A from porcine skin, 300 bloom, average molecular weight = 60 kDa), chitosan (from shrimp shells, average molecular weight = 150 kDa, degree of deacetylation = 85%) and chondroitin-6-sulfate (sodium salt from shark cartilage) were obtained from Sigma-Aldrich (St. Louis, MO‎, USA). Bloomage Freda Biopharm Co., Ltd. (Jinan, China). Provided sodium hyaluronan (average molecular weight = 1.3 MDa). 2-(*N*-morpholino) ethanesulfonic acid (MES), trypsin-EDTA and antibiotics were acquired from Sigma-Aldrich while 1-ethyl-3-(3-dimethylamino-propyl) carbodiimide (EDC) was obtained from Acros. Fetal bovine serum (FBS, HyClone, Logan, UT, USA) and Dulbecco’s Modified Eagle’s Medium/Nutrient Mixture F-12 (DMEM/F-12, Sigma, St. Louis, MO‎, USA) were used for cell culture. Cell staining used 4,6-diamidino-2-phenylindole (DAPI) solution (Life Technologies, Carlsbad, CA, USA).

### 4.2. Preparation of Cryogel

The cryogel used in the study was prepared following the procedure described before with some modification [15]. In brief, 6% (*w*/*v*) gelatin and 2% (*w*/*v*) chitosan were dissolved in MES buffer (pH 6, 0.5 M), heated and maintained at 70 °C for 2 h (solution A). A solution of 2% (*w*/*v*) chondroitin-6-sulfate and 0.1% (*w*/*v*) sodium hyaluronan was prepared separately in MES buffer (pH 6, 0.5 M) (solution B). The crosslinking agent EDC was added to solution B to a final concentration of 4% (*w*/*v*) and left to react for 30 min at 37 °C for activation of the carboxyl groups. Subsequently, 2 mL each of solution A and activated solution B were transferred to a 5 mL plastic syringe (inside diameter = 13 mm) and mixed in a shaking water bath at 70 °C for 30 s, before being immersed in 95% alcohol pre-equilibrated in a deep freezer at −16 °C and frozen for 16 h. At the end of the reaction, the syringe was removed from the freezer and thawed at room temperature. The formed cryogel was pushed out from the syringe and cut with a sharp blade and a biopsy puncher into disks (2 mm in thickness and 10 mm in diameter). The cryogel disks were sequentially washed with phosphate buffered saline (PBS) at room temperature and deionized distilled (DDI) water at 70 °C. The final weight percentage of the cryogel is 66.1% gelatin, 16.5% chondroitin-6-sulfate, 0.83% sodium hyaluronan and 16.5% chitosan.

### 4.3. Isolation and Cultivation of Porcine Chondrocytes and Adipose-Derived Stem Cells (ASCs)

The Institutional Animal Care and Use Committee of Chang Gung University approved animal experiments under IACUC Approval No. CGU13-035 (31/05/2013). Chondrocytes were harvested from the porcine knee articular cartilage as described previously [59]. After being sub-cultured for three passages, the cells were trypsinized and mixed with DMEM/F12. Porcine ASCs were harvested and isolated according to a modification of the procedures reported in the literature [60]. Briefly, the adipose tissue was harvested from the lower abdomen of the pig after sterilizing the lower abdomen with a β-iodine solution. The fat was diced into very small pieces using scissors in a sterile procedure. The diced fat tissue sample was washed extensively with equal volumes of PBS, and digested with 0.05% collagenase in a 37 °C water bath shaken at 165 rpm for 30 min. To obtain cell pellet at a high density, the enzyme reaction was stopped by adding an equal volume of DMEM for neutralization, followed by centrifugation for 10 min at 250× *g*. The supernatant was discarded, and the cell pellet was re-suspended in 160 mM NH_4_Cl and incubated at room temperature for 10 min to lyse the contaminating red blood cells. The cell pellet was re-collected by centrifugation as described above, filtered through a 100 μL nylon mesh to remove the cellular debris and incubated overnight in the control medium in culture dishes at 37 °C in a 5% CO_2_ environment. Following the incubation, the culture dishes were washed extensively with PBS to remove the residual non-adherent red blood cells, and the density fraction enriched with ASCs was collected.

### 4.4. Dynamic Culture of Chondrocytes in Cryogel Scaffolds

Chondrocytes grown after the second passage (P3) were used. Ten microliters of chondrocytes (10^7^ cell/mL) were seeded into cryogel scaffolds placed in a 48-well cell culture plate, which have been sterilized with 75% ethanol for 24 h followed by UV light exposure overnight in a laminar flow hood and saturated with the cell culture medium (DMEM/F12 containing 10% FBS and 1.0% streptomycin–penicillin solution). After 4 h for cell attachment, the cells/scaffold constructs were transferred to a new well and cultured with 2 mL of the cell culture medium in a 37 °C CO_2_ incubator. Next, the construct was placed in a chamber of a mechanical-driven multi-specimen bioreactor (ElectroForce BioDynamic 5200, TA Instruments, New Castle, DE, USA), which has four chambers set on a single-axis (compression) configuration with a 200 N load cell. Each chamber was filled with 150 mL of cell culture medium and dynamic culture was carried out in the bioreactor for 14 days using with the following settings: frequency = 1 Hz; strain = 10%, 20%, or 40%; stimulation duration = 1, 3, or 9 h/day. The experiment groups under dynamic culture with 10% (20%) strain and 1 h/day (3 h/day) stimulation duration are termed D11 (D23) etc. Static culture in the 48-well cell culture plate was used as a control.

### 4.5. Dynamic Culturing of ASCs/Chondrocytes in Cryogel Scaffolds

For co-culture of ASCs/chondrocytes, we followed the same procedure as described above for cryogel scaffold preparation, cell seeding and cell culture. The cells were mixed at 1:1 cell number ratio (total cell number = 1 × 10^5^) and then seeded into the scaffolds. Mono-culture with 1 × 10^5^ ASCs (P2) or chondrocytes (P3) were used for comparison. The bioreactor’s settings were based on the optimal strain and stimulation duration obtained from the dynamic culture of chondrocytes, i.e., strain = 20% and duration = 3 h/day. The constructs were cultured in the bioreactor at a frequency of 1 Hz for 14 days before gene expression and biochemical analysis. Static culture in the 48-well cell culture plate was used as a control for each group.

### 4.6. Gene Expression

To examine cartilage-specific marker gene expression, we used standard protocols for RNA isolation and cDNA synthesis [61]. Quantitative real-time polymerase chain reaction (qRT-PCR) measurements were performed using a SYBR Green RT-PCR kit (SYBR Green I supermix, Bio-Rad, Hercules, CA, USA) in a MiniOpticon™ real-time PCR detection system (Bio-Rad CFD-3120). The primers used in the experiment are listed in Table 1. β-actin acted as a housekeeping control. Type I collagen (Col I), type II collagen (Col II), type X collagen (Col X), tumor necrosis factor-α (TGF-α), transforming growth factor-β1 (TGF-β1) and insulin-like growth factor-1 (IGF-1) genes were selected for analysis.

### 4.7. Biochemical Analysis

One milliliter of digestion solution (55 mM sodium citrate, 150 mM sodium chloride, 5 mM cysteine HCl, 5 mM EDTA and 0.2 mg/mL papain) was added to the cells/scaffold constructs in the celll culture plate and shaken at 60 °C for 24 h to before determining the DNA, glycoaminoglycans (GAGs) and Col II contents. The DNA content was determined with bisBenzimide H 33258 solution using an enzyme-linked immunosorbent assay (ELISA) reader (Excitation: 365 nm, Emission: 458 nm) [62]. For GAGs content, 1,9-dimethylmethylene blue was used with shark cartilage chondroitin-6-sulfate as the standard in an ELISA reader at a wavelength of 525 nm [63]. The quantitative determination of Col II content was by sandwich ELISA assays using rabbit anti-Col II polyclonal antibody (Bioss bs-0709R, Woburn, MA, USA) and HRP-conjugated rabbit anti-Col II polyclonal antibody (Bioss bs-0709R-HRP) in an ELISA reader at 450 nm with chicken Col II as the standard.

### 4.8. Immunofluorescence Staining

The cells/scaffold constructs were soaked in 10% formaldehyde solution overnight at 4 °C and washed in PBS containing 0.1% Tween 20 (PBST). The washed constructs were soaked in a blocking buffer for 1 min before another wash with PBST for another 30 min. Subsequently, rabbit anti-gelatin primary antibody (MyBioSource MBS625052, San Diego, CA, USA) was allowed to react with gelatin in the cryogel scaffold for 2 h, followed by anti-rabbit IgG-FITC secodary abtibody (Jackson ImmunoResearch Laboratories Inc. 711-095-152, West Grove, PA, USA) for 1 h. Mouse anti-Col II primary antibody (LSBio LS-C128248, Seattle, WA, USA) was added and incubated overnight at 4 °C. The constructs were washed three times in PBST, and then incubated in anti-mouse IgG-Cy3 secondary anti-body (Jackson ImmunoResearch Laboratories Inc. 115-165-003). After washing three times in PBST, the construct was incubated for additional 60 min at room temperature in 50 mg/mL DAPI for nuclear staining and washed three times in PBST for 30 min. The fluorescence-stained cells were onserved with a Zeiss LSM 510 Meta confocal laser-scanning microscope (Oberkochen, Germany) for blue (DAPI), green (FITC) and red (Cy3) fluorescence. The excitation wavelength was 364/488/543 nm and the emission wavelength was 407–482/500–550/550–650nm (blue/green/red).

### 4.9. In Vivo Subcutaneous Implantation

A subcutaneous implantation protocol was further used to evaluate biocompatibility. All animal procedures were approved by the Institutional Animal Care and Use Committee of Chang Gung University (IACUC Approval No.: CGU13-140, 18/03/2014). The National Laboratory Animal Center (Taipei, Taiwan) provided male 6-week old nude mice (BALB/cAnN.Cg-Foxn1nu/CrlNarl) for the in vivo animal studies, which were housed in the Laboratory Animal Center of Chang Gung University. Cells/scaffold constructs after static or dynamic culture for 14 days were implanted subcutaneously within the dorsal area of the nude mice. After 8 weeks, we retrieved the constructs and fixed them in 4% paraformaldehyde prepared in 100 mM sodium cacodylate buffer (pH 7.4) at 48 °C overnight. This was followed by dehydration in graded ethanol solution, embedding in paraffin, sectioning and mounting on microscope slides. The specimens were subject to Alcian blue (1% Alcian blue + hematoxylin solution) and Safranin O (1.0% Safranin O + hematoxylin) staining following standard protocols. Immunohistochemical staining was performed using mouse anti-Col II (LSBio LS-C128248) primary antibody followed with the UltraVision Quanto Detection System HRP DAB (Thermo Fisher Scientific, Waltham, MA, USA) [55].

### 4.10. Statistical Analysis

All data are expressed as mean ± standard deviation. Statistical significance was declared when the *p* value was less than 0.05. One-way analysis of variance (ANOVA) was used for statistical analysis between groups. Tukey’s post-hoc test was used to compare pairs of groups if significance was found.

## 5. Conclusions

This study successfully identified the best dynamic culture condition to be 20% strain and 3 h/day stimulation duration during cyclic dynamic compressive loading of chondrocytes in cryogel scaffolds at 1 Hz. Under this setting, chondrocytes in the highly elastic gelatin/chondroitin sulfate/hyaluronan/chitosan cryogel properly showed an enhanced chondrogenic phenotype from gene expression and biochemical analyses. This study also demonstrated that mechanical stimulation could promote the effectiveness of ASCs’ differentiation toward the chondrogenic lineage by co-culture chondrocytes with ASCs in vitro. Furthermore, the co-cultured cells/scaffold constructs could be implanted in vivo for neo-cartilage formation. Considering the tissue engineering approach for cartilage regeneration, outcomes from this study suggested the possibility to reduce the amount of donor cartilage needed by substituting chondrocytes with ASCs and by supplementing with in vitro mechanical stimulation before implantation. This practice could also prevent the use of growth factors, which is a notable clinical advantage.

## Figures and Tables

**Table 1:** Primer sequences for quantitative real-time polymerase chain reaction (qRT-PCR) analysis of gene expression.

Gene	Primer Sequence	
*β-actin*	forward	AAGCCAACCGTGAGAAGATG
	reverse	GTACATGGCTGGGGTGTTG
*Col I*	forward	CCAACAAGGCCAAGAAGAAG
	reverse	ATGGTACCTGAGGCCGTTCT
*Col II*	forward	GCACGGATGGTCCCAAAG
	reverse	CAGCAGCTCCCCTCTCAC
*Col X*	forward	CACCAAGGCACAGTTCTTCA
	reverse	ACCGGAATACCTTGCTCTC
*TNF-α*	forward	CCCTTCCACCAACGTTTTCCT
	reverse	TGATGGCAGAGAGGAGGTTG
*TGF-β1*	forward	TTTCGCCTCAGTGCCCA
	reverse	GCCAGAATTGAACCCGTTAA
*IGF-1*	forward	TTCGCATCTCTTCTACTTGGCCCT
	reverse	CGTACCCTGTGGGCTTGTTGAAAT

## Abbreviations

3D	Three-dimensional
ECM	Extracellular matrix
Col II	Type II collagen
Col I	Type I collagen
Col X	Type X collagen
TNF-α	Tumor necrosis factor-α
IGF-1	Insulin growth factor-1
TGF-β1	Transforming growth factor-β1
BMPs	Bone morphogenetic proteins
GAGs	Glycoaminoglycans
ASCs	Adipose-derived stem cells
MSCs	Mesenchymal stem cells
DAPI	4′,6-diamidino-2-phenylindole
FITC	Fluorescein isothiocyanate
Cy 3	Cyanine 3
RGD	Arg-Gly-Asp
PEG	Polyethylene glycol
qRT-PCR	Quantitative real-time polymerase chain reaction
